# Geriatric symptoms associated with healthy life expectancy in older people in Japan

**DOI:** 10.1265/ehpm.22-00300

**Published:** 2023-07-07

**Authors:** Rikuya Hosokawa, Toshiyuki Ojima, Tomoya Myojin, Katsunori Kondo, Naoki Kondo

**Affiliations:** 1 Department of Human Health Sciences, Graduate School of Medicine, Kyoto University, Kyoto 606-8507, Japan; 2 Department of Community Health and Preventive Medicine, Hamamatsu University School of Medicine, Shizuoka 431-3192, Japan; 3 Department of Public Health, Health Management and Policy, Nara Medical University, Nara 634-8521, Japan; 4 Center for Preventive Medical Sciences, Chiba University, Chiba 263-8522, Japan; 5 Center for Well-being and Society, Nihon Fukushi University, Aichi 470-3295, Japan; 6 Center for Gerontology and Social Science, Research Institute, National Center for Geriatrics and Gerontology, Aichi 474-8511, Japan; 7 School of Public Health and Graduate School of Medicine, Kyoto University, Kyoto 606-8507, Japan

**Keywords:** Geriatric symptoms, Healthy life expectancy, Older people

## Abstract

**Background:**

We investigated the relationship between characteristic geriatric symptoms and healthy life expectancy in older adults in Japan. Additionally, we determined relationship predictors that would help formulate effective approaches toward promoting healthy life expectancy.

**Methods:**

The Kihon Checklist was used to identify older people at high risk of requiring nursing care in the near future. We evaluated the association of geriatric symptoms with healthy life expectancy while considering risk factors (frailty, poor motor function, poor nutrition, poor oral function, confinement, poor cognitive function, and depression). Data from the 2013 and 2019 Japan Gerontological Evaluation Studies were used. Healthy life expectancy was assessed using the multistate life table method.

**Results:**

Overall, 8,956 individuals were included. For both men and women, healthy life expectancy was shorter in the symptomatic group than in the asymptomatic group for several domains of the Kihon Checklist. For men, the difference between individuals with risk factors and those with no risk factors was the maximum for confinement (3.83 years) and the minimum for cognitive function (1.51 years). For women, the difference between individuals with risk factors and those with no risk factors was the maximum for frailty (4.21 years) and the minimum for cognitive function (1.67 years). Healthy life expectancy tended to be shorter when the number of risk factors was higher. Specifically, the difference between individuals with ≥3 risk factors and those with no risk factors was 4.46 years for men and 5.68 years for women.

**Conclusions:**

Healthy life expectancy was negatively associated with characteristic geriatric symptoms, with strong associations with frailty, physical functional decline, and depression. Therefore, comprehensive assessment and prevention of geriatric symptoms may increase healthy life expectancy.

**Supplementary information:**

The online version contains supplementary material available at https://doi.org/10.1265/ehpm.22-00300.

## Background

In Japan, the proportion of older adults continues to increase as the longevity of society increases. The Japanese population has the world’s longest average life expectancy, and statistics demonstrate an increase in survival years [1]. In addition, life expectancy is continuously increasing in most countries [2, 3]. Longer periods of disability are associated with greater cost of medical care and other social security expenses [4, 5]. With the aging population, the quality of life (QOL), not just life expectancy, is gaining more attention. Healthy life expectancy is defined as the average number of years that a person can live in a healthy state and is an index of mortality and health status [6, 7]. Healthy life expectancy captures both the quantity and quality of years lived [8, 9]. In Japan, “Healthy Japan 21” is a national policy that focuses on extending healthy life expectancy beyond the total number of years lived and proposes compressing the period spent in unhealthy conditions [10]. Moreover, the World Health Organization promotes a strategy that considers the expansion of healthy life expectancy an important goal [11].

Life expectancy captures the mortality rate over the entire life course [12]. Adequate medical care, nursing care services, and social security are crucial for better QOL. Japan has achieved the longest life expectancy in the world and a high level of healthy life expectancy [13, 14]. As of 2016, in Japan, the average life expectancy was 87.14 and 80.98 years for women and men, respectively and healthy life expectancy was 74.79 and 72.14 years for women and men, respectively [15]. The average life expectancy and healthy life expectancy of Japanese people are increasing; however, there remains a gap of approximately 10 years between average life expectancy and healthy life expectancy that needs to be addressed. Healthy life expectancy, which is achieved by eliminating chronic diseases and injuries, has been proposed as an indicator of disease burden [16, 17]. To improve healthy life expectancy, characteristic geriatric symptoms, which are also considered risk factors for reduced healthy life expectancy in older adults, must be assessed.

With the rapid aging of the population, a long-term care insurance (LTCI) system has been implemented in Japan [18, 19]. The LTCI is a social insurance system that assists frail and disabled older people in their daily activities. People aged older than 65 years who need care are candidates for formal care services, which they can receive based on the criteria set by the government. Japan has a basic checklist of self-administered questionnaires used for a comprehensive functional assessment of older adults to identify those who are at high risk of requiring nursing care in the near future. The Ministry of Health, Labour and Welfare recommends the use of the Kihon Checklist (KCL) to screen the target population requiring nursing care prevention and LTCI services and evaluate the effectiveness of interventions [20–23]. The KCL was developed to identify the health challenges posed by comprehensive geriatric symptoms, including physical, cognitive, and sociopsychological problems, in the older population. It is a self-assessment tool consisting of 25 Yes/No items divided into seven domains: activities of daily living, physical activity, nutritional status, oral function, confinement, cognitive status, and depression status. The KCL is effective in predicting the future incidence of frailty status, care dependence, and mortality; furthermore, the advantages of the KCL are that the 25 items are simple (i.e., the response options are yes or no). The items are organized in domains, which allows researchers to easily identify problems that suggest a need for intervention [24–27]. However, the relationship between characteristic geriatric symptoms and healthy life expectancy has not been fully elucidated. Therefore, there is a need to understand the relationship between the characteristics of geriatric symptoms and healthy life expectancy and the associated predictors that can help develop approaches to promote healthy life expectancy. This study aimed to clarify the relationship between characteristic geriatric symptoms and healthy life expectancy of older adults in Japan.

## Methods

**Fig. 1:**
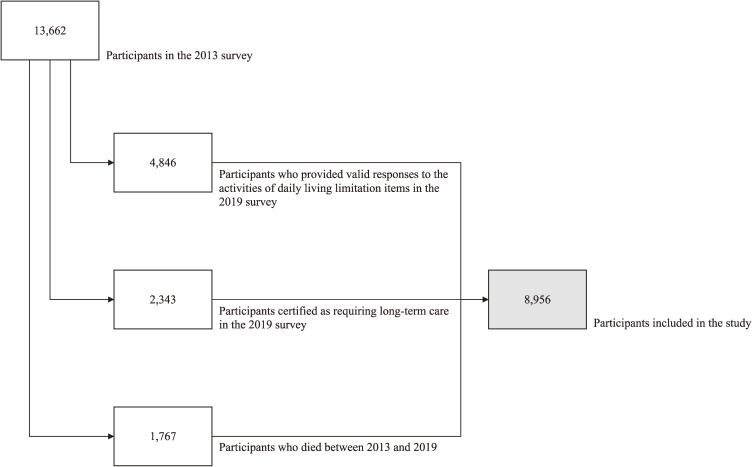
Flowchart of participant inclusion in this study

The present study used nationwide longitudinal cohort data from the Japan Gerontological Evaluation Study [28, 29] from two time points: 2013 and 2019. The study focused on older adults aged ≥65 years in Japan with the aim of achieving a healthy society with longevity. This cohort study investigated health factors and social, environmental, and behavioral factors related to functional decline and cognitive impairment. Residents aged ≥65 years who were not certified as requiring long-term care from 19 municipalities of 9 of the 47 prefectures throughout Japan were included. The survey was conducted using a self-administered questionnaire. The main survey items were related to the risk of care and questions on activities of daily living. Of the 13,662 participants who provided valid responses to the activities of daily living limitation items in the 2013 survey, we included 4,846 participants who provided valid responses to the activities of daily living limitation items in the 2019 survey. In addition, we included 2,343 participants who were certified as requiring long-term care in the 2019 survey and 1,767 people who died between 2013 and 2019. Therefore, the total number of participants was 8,956 (Fig. 1).

### Exposure: characteristic geriatric symptoms

The physical, psychological, functional, and social conditions of older adults were comprehensively assessed using the KCL (Additional file 1) [20–23]. The KCL is a self-administered questionnaire that requires a respondent to answer “Yes” or “No” to 25 questions related to living conditions and physical and mental functioning. The KCL has already been translated into English and Portuguese and is used in several countries [30–32]. This self-report questionnaire consists of a set of questions in seven domains: activities of daily living, physical activity, nutritional status, oral function, confinement, cognitive status, and depression status. In this study, judgments made based on the KCL were mainly based on the following criteria: it determines “frailty” from the items on activities related to daily living, “decline in physical activity” from the items on motor function, “decline in nutritional state” from the items on nutritional status, “decline in oral function” from the items on oral function, “confinement” from the items on confinement, “decline in cognitive function” from the items on cognitive function, and “depressed mood” from the items on depressed mood. Furthermore, the above items were tabulated as “no risk factor,” “one risk factor,” “two risk factors,” and “three or more risk factors.”

### Outcome: healthy and unhealthy life expectancy

Healthy and unhealthy life expectancy were calculated from the multistate life table method using SAS stochastic population analysis for complex events (SPACE) (SAS Institute Inc., NC, USA) [33]. SPACE consists of SAS with SAS/IML and SAS/Connect. For more technical information on the SPACE program, please refer to the study by Cai et al. [33]. In the current analysis, the Markov transition model for disability and death consisted of three states: two non-absorbing states (non-disability and disability) and one absorbing state (death).

There are four possible health-state transitions over time: from non-disabled to disabled (occurrence of disability), from disabled to non-disabled (recovery of disability), from non-disabled to death, and from disabled to death. The transition in the status was based on changes from 2013 at baseline to 2019 at follow-up: no disability was estimated as being healthy, and having a disability was estimated as being unhealthy. Based on this estimated transition rate, the study calculated healthy life expectancy at the age of 65 years for men and women. In this study, disability was determined from answers to the questions about disability in daily life at baseline and follow-up, and the data on LTCI care needs were obtained from the municipalities. Death was also determined based on the data obtained from the municipalities.

### Statistical analyses

We examined the differences in healthy life expectancy depending on the presence or absence of the following seven risk factors in the KCL: frailty, physical activity, nutritional status, oral function, confinement, cognitive function, and depressive mood. In addition, we examined the difference that occurred in healthy life expectancy according to the number of risk factors (specifically, “no risk factors,” “one risk factor,” “two risk factors,” and “three or more risk factors”). Significant differences were determined at 95% confidence intervals.

### Ethical statement

Ethical approval for this study was obtained from Nihon Fukushi University (approval number: 13-14), Chiba University (approval number: 2493), and National Institute for Longevity Sciences (approval number: 992-3). This study was conducted in accordance with the principles of the Declaration of Helsinki. All participants were informed that participation in this study was voluntary and that completing the questionnaire, selecting the checkbox for consent, and returning it by mail constituted consent to participate in the study. We considered the return of the completed questionnaire as consent to participate in the study, including baseline survey data and follow-up. In addition, information on LTCI certification status and death was confirmed after written consent was obtained.

## Results

### Participant characteristics

Table 1 presents the characteristics of the study participants. Overall, 8,956 participants were included, comprising 4,348 men (mean age: 74.3 years [standard deviation, 6.2]) and 4,608 women (mean age: 74.5 years [standard deviation, 6.4]). At baseline (in 2013), there were 3,624 (83.3%) active and 724 (16.7%) disabled men and 3,901 (84.7%) active and 707 (15.3%) disabled women. At the follow-up (in 2019), there were 2,262 (52.0%) active, 942 (21.7%) disabled, and 1,144 (26.3%) deceased men; and 2,548 (55.3%) active, 1,437 (31.2%) disabled, and 623 (13.5%) deceased women.

**Table 1:** Characteristics of patients in the analysis sample

		**Men**		**Women**	

		**N**	**%**	**N**	**%**
Age (2013)	65–74 years	2,429	55.9	2,544	55.2
	≥75 years	1,919	44.1	2,064	44.8

	Mean, SD	74.3	6.2	74.5	6.4

Health status (2013)	Active	3,624	83.3	3,901	84.7
	Disabled	724	16.7	707	15.3

Health status (2019)	Active	2,262	52.0	2,548	55.3
	Disabled	942	21.7	1,437	31.2
	Death	1,144	26.3	623	13.5

Total		4,348	100.0	4,608	100.0

Note: SD, standard deviation

Table 2 shows data related to healthy life expectancy, which was 16.18 years (95% confidence interval [CI], 15.78–16.57) for men and 16.97 years (95% CI, 16.53–17.41) for women at the age of 65 years.

**Table 2:** Healthy life expectancy and unhealthy life expectancy at the age of 65 years

	**Healthy life expectancy**			**Unhealthy life expectancy**		

	**Years**	**95% CI**		**Years**	**95% CI**	
Men	16.18	15.78	16.57	5.07	4.60	5.54
Women	16.97	16.53	17.41	10.60	9.63	11.56

Note: Healthy life expectancy vs. unhealthy life expectancy at 65 years of age.

CI, confidence interval.

### Characteristic geriatric symptoms

The distribution of each characteristic geriatric symptom among men was as follows (Table 3): frailty, 152 (3.5%); decline in physical activity, 600 (13.8%); decline in nutritional state, 76 (1.7%); decline in oral function, 696 (16.0%); confinement, 184 (4.2%); decline in cognitive function, 1,563 (35.9%); and depressive mood, 1,055 (24.3%). The characteristics for women were as follows: frailty, 196 (4.3%); decline in physical activity, 1,051 (22.8%); decline in nutritional state, 87 (1.9%); decline in oral function, 668 (14.5); confinement, 173 (3.8%); decline in cognitive function, 1,548 (33.6%); and depressive mood, 1,098 (23.8%).

**Table 3:** Characteristic geriatric symptoms

		**Men**		**Women**	

		**N**	**%**	**N**	**%**
Frailty	No	4,196	96.5	4,412	95.7
	Yes	152	3.5	196	4.3

Decline in physical activity	No	3,748	86.2	3,557	77.2
	Yes	600	13.8	1,051	22.8

Decline in nutritional state	No	4,272	98.3	4,521	98.1
	Yes	76	1.7	87	1.9

Decline in oral function	No	3,652	84.0	3,940	85.5
	Yes	696	16.0	668	14.5

Confinement	No	4,164	95.8	4,435	96.2
	Yes	184	4.2	173	3.8

Decline in cognitive function	No	2,785	64.1	3,060	66.4
	Yes	1,563	35.9	1,548	33.6

Depressed mood	No	3,293	75.7	3,510	76.2
	Yes	1,055	24.3	1,098	23.8

The distribution of the total number of characteristic geriatric symptoms is represented in Table 4. Overall, 1,295 (29.8%) men had one symptom, 641 (14.7%) had two symptoms, and 478 (11.0%) had three or more symptoms. Meanwhile, 1,331 (28.9%) women had one symptom, 708 (15.4%) had two symptoms, and 570 (12.4%) had three or more symptoms.

**Table 4:** Number of characteristic geriatric symptoms

	**Men**		**Women**	

	**N**	**%**	**N**	**%**
No symptoms	1,934	44.5	1,999	43.4
One symptom	1,295	29.8	1,331	28.9
Two symptoms	641	14.7	708	15.4
Three or more symptoms	478	11.0	570	12.4

### Association between characteristic geriatric symptoms and healthy life expectancy

The relationship between each geriatric symptom and healthy life expectancy in men is presented in Table 5. Risk-free men tended to have a longer healthy life expectancy than at-risk men in six domains. In particular, the at-risk group had a shorter healthy life expectancy than the risk-free group: frailty, 3.12 years; decline in physical activity, 3.49 years; decline in oral function, 1.77 years; confinement, 3.83 years; decline in cognitive function, 1.51 years; and depressive mood, 2.60 years.

**Table 5:** Association between characteristic geriatric symptoms and healthy life expectancy in men

		**Healthy life expectancy**			**Unhealthy life expectancy**		

		**Years**	**95% CI**		**Years**	**95% CI**	
Frailty	No	16.25	15.86	16.64	5.22	4.71	5.74
	Yes	13.13	11.12	15.13	6.10	4.29	7.92

Decline in physical activity	No	16.53	16.13	16.93	5.09	4.54	5.64
	Yes	13.04	11.95	14.12	6.84	5.66	8.02

Decline in nutritional state	No	16.22	15.82	16.61	5.14	4.65	5.63
	Yes	13.62	11.35	15.90	4.85	2.55	7.15

Decline in oral function	No	16.41	15.99	16.84	5.10	4.58	5.62
	Yes	14.64	13.84	15.45	5.73	4.78	6.67

Confinement	No	16.29	15.89	16.70	5.21	4.71	5.71
	Yes	12.46	10.83	14.10	6.09	4.39	7.78

Decline in cognitive function	No	16.68	16.24	17.12	4.75	4.17	5.32
	Yes	15.17	14.52	15.83	5.81	5.06	6.55

Depressed mood	No	16.76	16.33	17.19	5.77	5.04	6.51
	Yes	14.16	13.35	14.96	5.25	4.46	6.04

Note: Healthy life expectancy and unhealthy life expectancy at 65 years of age.

CI, confidence interval.

The relationship between the total number of geriatric symptoms and healthy life expectancy (Table 6) showed that healthy life expectancy tended to be shorter when the number of risk factors was higher. Specifically, three or more risk factors were associated with a healthy life expectancy of 13.05 years (95% CI, 11.88–14.22), while no risk factors were associated with a healthy life expectancy of 17.51 years (95% CI, 16.93–18.08), a difference of more than 4 years.

**Table 6:** Association between number of characteristic geriatric symptoms and healthy life expectancy in men

	**Healthy life expectancy**			**Unhealthy life expectancy**		

	**Years**	**95% CI**		**Years**	**95% CI**	
No symptoms	17.51	16.93	18.08	5.63	4.48	6.78
One symptom	15.86	15.19	16.53	5.87	4.80	6.94
Two symptoms	14.07	13.14	15.00	6.14	4.99	7.28
Three or more symptoms	13.05	11.88	14.22	6.38	5.28	7.49

Note: Healthy life expectancy and unhealthy life expectancy at 65 years of age.

CI, confidence interval.

The relationship between each of the characteristic geriatric symptoms and healthy life expectancy in women is presented in Table 7. The at-risk group had a shorter healthy life expectancy than that of the risk-free group, with differences as follows: frailty, 4.21 years; decline in physical activity, 3.15 years; decline in oral function, 2.54 years; decline in cognitive function, 1.67 years; and depressive mood, 3.12 years.

**Table 7:** Association between characteristic geriatric symptoms and healthy life expectancy in women

		**Healthy life expectancy**			**Unhealthy life expectancy**		

		**Years**	**95% CI**		**Years**	**95% CI**	
Frailty	No	17.06	16.62	17.50	10.88	9.81	11.95
	Yes	12.85	10.77	14.92	13.38	10.77	15.99

Decline in physical activity	No	17.57	17.06	18.08	11.72	10.19	13.26
	Yes	14.42	13.56	15.28	11.82	10.46	13.18

Decline in nutritional state	No	17.01	16.58	17.45	10.66	9.68	11.64
	Yes	14.55	11.50	17.60	9.93	5.30	14.57

Decline in oral function	No	17.29	16.83	17.76	10.81	9.67	11.95
	Yes	14.75	13.63	15.87	11.74	9.91	13.58

Confinement	No	17.00	16.56	17.44	11.33	10.10	12.56
	Yes	15.30	13.12	17.47	10.20	7.92	12.47

Decline in cognitive function	No	17.48	16.96	18.01	10.05	8.87	11.24
	Yes	15.81	15.08	16.55	11.85	10.55	13.15

Depressed mood	No	17.64	17.13	18.14	11.02	9.68	12.36
	Yes	14.52	13.70	15.33	11.83	10.51	13.14

Note: Healthy life expectancy and unhealthy life expectancy at 65 years of age.

CI, confidence interval.

The relationship between the overall characteristic geriatric symptoms and healthy life expectancy is shown in Table 8. The relationship between the total number of geriatric symptoms and healthy life expectancy showed that healthy life expectancy was shorter for individuals with more risk factors. Specifically, three or more risk factors were associated with a life expectancy of 12.90 years (95% CI, 11.60–14.20), while no risk factors were associated with a life expectancy of 18.58 years (95% CI, 17.91–19.25), a difference of more than 5 years.

**Table 8:** Association between number of characteristic geriatric symptoms and healthy life expectancy in women

	**Healthy life expectancy**			**Unhealthy life expectancy**		

	**Years**	**95% CI**		**Years**	**95% CI**	
No symptoms	18.58	17.91	19.25	13.27	10.29	16.26
One symptom	16.65	15.81	17.49	10.98	9.22	12.75
Two symptoms	14.99	14.03	15.95	12.59	10.62	14.57
Three or more symptoms	12.90	11.60	14.20	13.28	11.51	15.05

Note: Healthy life expectancy and unhealthy life expectancy at 65 years of age.

CI, confidence interval.

## Discussion

To the best of our knowledge, this is the first study to investigate the relationship between comprehensive geriatric symptoms and healthy life expectancy in older adults. The results showed that healthy life expectancy tended to be shorter in the at-risk group than in the non-at-risk group in several domains assessed using the KCL: frailty, poor motor function, poor oral function, confinement, poor cognitive function, and depression.

Frailty, poor motor function, poor oral function, confinement, poor cognitive function, and depression were associated with healthy life expectancy. Among these factors, frailty, poor motor function, and depression were particularly associated with a health span that was shorter by more than 3 years in the at-risk group than in the non-at-risk group. This may be due to the fact that frailty, poor motor function, and depression put individuals at a greater risk of requiring long-term care and developing disabilities [34–37]. Additionally, the healthy life expectancy per item between men and women all showed a trend toward women having a longer life expectancy than men. This difference may be due to differences in life expectancy between the sexes [1]. Frailty is a series of clinical symptoms associated with aging that indicates a decline in physical fitness and physiological discomfort [38, 39]. Frailty in older people is likely to worsen [40, 41]. It has also been reported to increase the risk of adverse health outcomes, including long-term care and mortality [42, 43]. Nevertheless, frailty is a reversible and dynamic condition with potential for improvement and progression [44]. Therefore, establishing appropriate interventions for frailty may be beneficial. Furthermore, this study was conducted before the coronavirus disease-2019 (COVID-19) pandemic; however, since COVID-19, there has been an increase in confinement among older individuals, raising concern that the association may be even stronger at present [45, 46].

Regarding mental health, life expectancy of men with depression was shorter by 2.60 years compared to that of those without, while the life expectancy of women with depression was shorter by 3.12 years compared to that of those without. Other studies have shown that depression can affect health in several ways, including the development of several chronic diseases [47–49]. Depression is also associated with poor health-related QOL and increases the number of years lived with disability [50]. Depression is potentially life-threatening and is associated with mortality and reduced life expectancy [51–53]. Therefore, depression may negatively influence healthy life expectancy.

Moreover, compared with no risk factors, three or more risk factors tended to reduce healthy life expectancy. For instance, individuals who are physically active or do not have factors that prevent them from maintaining an appropriate weight may improve their health and increase their life expectancy compared to those with multiple risk factors [54]. This suggests that a higher number of more complex risk factors, including frailty, poor motor function, poor oral function, confinement, poor cognitive function, and depression, is associated with a shorter healthy life expectancy.

### Limitations

This study has some limitations. First, this study may not be completely free from detection bias because not all data for participants regarding activities of daily living limitation, as determined by responses to the questions on activities of daily living limitation at baseline and the data on LTCI care requirements obtained from the local government, were included for LTCI certification. Second, the estimated life expectancy at the age of 65 years in this study was 21.25 years for men and 27.57 years for women (Table 2). Conversely, the life expectancy at the age of 65 years reported by the government for the same period was 19.57 years for men and 24.43 years for women [55]. Both male and female participants had a higher estimated life expectancy than the general population; thus, these participants may have been healthier than the general population. Therefore, the association between risk factors and healthy life expectancy may have been underestimated. Finally, this observational study revealed the association between geriatric symptoms and healthy life expectancy in older adults; however, it did not show a causal relationship. Future research should elucidate the causal relationship through intervention studies.

## Conclusions

Characteristic geriatric symptoms were found to be negatively associated with healthy life expectancy. In particular, frailty, physical functional decline, and depression were strongly associated with healthy life expectancy. In addition, as the overall risk factors increased, healthy life expectancies tended to decrease. Therefore, comprehensive assessment and prevention of geriatric symptoms may increase longevity.

## Abbreviations

CI: confidence interval
COVID-19: coronavirus disease-2019
KCL: Kihon Checklist
LTCI: long-term care insurance
QOL: quality of life
SPACE: stochastic population analysis for complex events

## Supplementary information

**Additional file 1:** Kihon Checklist. The table contained in this additional file presents the Kihon Checklist, a set of 25 questions designed to assess the ability of older adults to perform their activities of daily living. Each question has a binary response (Yes/No) and a score is assigned to each response.

## Declarations

### Availability of data and materials

Data are available from the authors upon request.
