# Supplementary material for: Geriatric symptoms associated with healthy life expectancy in older people in Japan
Source: Environ Health Prev Med. 2023 Jul 7;28:44. doi: 10.1265/ehpm.22-00300 (PMC10331002; doi:10.1265/ehpm.22-00300)
Supplement: Supplementary file 1 — Additional file 1: Kihon Checklist. The table contained in this additional file presents the Kihon Checklist, a set of 25 questions designed to assess the ability of older adults to perform their activities of daily living. Each question has a binary response (Yes/No) and a score is assigned to each response. [file ehpm-28-044-s001.docx]

**Additional file 1. Kihon Checklist**

| No. | Question items | Score |
| --- | --- | --- |
| Q1 | Do you go out by bus or train by yourself? | [Yes = 0, No = 1] |
| Q2 | Do you go shopping to buy daily necessities by yourself? | [Yes = 0, No = 1] |
| Q3 | Do you manage your own deposits and savings at the bank? | [Yes = 0, No = 1] |
| Q4 | Do you sometimes visit your friends? | [Yes = 0, No = 1] |
| Q5 | Do you turn to your family or friends for advice? | [Yes = 0, No = 1] |
| Q6 | Do you normally climb stairs without using handrail or wall for support? | [Yes = 0, No = 1] |
| Q7 | Do you normally stand up from a chair without any aids? | [Yes = 0, No = 1] |
| Q8 | Do you normally walk continuously for 15 min? | [Yes = 0, No = 1] |
| Q9 | Have you experienced a fall in the past year? | [Yes = 1, No = 0] |
| Q10 | Do you have a fear of falling while walking? | [Yes = 1, No = 0] |
| Q11 | Have you lost 2 kg or more in the past 6 months? | [Yes = 1, No = 0] |
| Q12 | Height: cm, weight: kg, BMI: kg/m^2^ If BMI is less than 18.5, this item is scored. | [Yes = 1, No = 0] |
| Q13 | Do you have any difficulties eating tough foods compared to 6 months ago? | [Yes = 1, No = 0] |
| Q14 | Have you choked on your tea or soup recently? | [Yes = 1, No = 0] |
| Q15 | Do you often experience having a dry mouth? | [Yes = 1, No = 0] |
| Q16 | Do you go out at least once a week? | [Yes = 0, No = 1] |
| Q17 | Do you go out less frequently compared to last year? | [Yes = 1, No = 0] |
| Q18 | Do your family or friends point out your memory loss? e.g. “You ask the same question over and over again.” | [Yes = 1, No = 0] |
| Q19 | Do you make a call by looking up phone numbers? | [Yes = 0, No = 1] |
| Q20 | Do you find yourself not knowing today’s date? | [Yes = 1, No = 0] |
| Q21 | In the last 2 weeks, have you felt a lack of fulfillment in your daily life? | [Yes = 1, No = 0] |
| Q22 | In the last 2 weeks, have you felt a lack of joy when doing the things you used to enjoy? | [Yes = 1, No = 0] |
| Q23 | In the last 2 weeks, have you felt difficulty in doing what you could do easily before? | [Yes = 1, No = 0] |
| Q24 | In the last 2 weeks, have you felt helpless? | [Yes = 1, No = 0] |
| Q25 | In the last 2 weeks, have you felt tired without a reason? | [Yes = 1, No = 0] |

BMI, body mass index.
Note: A score of “1” for a Kihon checklist (KCL) item indicates difficulty in performing the activity in question, and a high total KCL score indicates an increased likelihood of requiring support to perform activities of daily living. The domains of the KCL are as follows: activities of daily living (Q1 to Q20), physical activity (Q6 to Q10), nutritional status (Q11 and Q12), oral function (Q13 to Q15), confinement (Q16 to Q17), cognitive status (Q18 to Q20), and depression status (Q21 to Q25).
